# ChatGPT vs. neurologists: a cross-sectional study investigating preference, satisfaction ratings and perceived empathy in responses among people living with multiple sclerosis

**DOI:** 10.1007/s00415-024-12328-x

**Published:** 2024-04-03

**Authors:** Elisabetta Maida, Marcello Moccia, Raffaele Palladino, Giovanna Borriello, Giuseppina Affinito, Marinella Clerico, Anna Maria Repice, Alessia Di Sapio, Rosa Iodice, Antonio Luca Spiezia, Maddalena Sparaco, Giuseppina Miele, Floriana Bile, Cristiano Scandurra, Diana Ferraro, Maria Laura Stromillo, Renato Docimo, Antonio De Martino, Luca Mancinelli, Gianmarco Abbadessa, Krzysztof Smolik, Lorenzo Lorusso, Maurizio Leone, Elisa Leveraro, Francesca Lauro, Francesca Trojsi, Lidia Mislin Streito, Francesca Gabriele, Fabiana Marinelli, Antonio Ianniello, Federica De Santis, Matteo Foschi, Nicola De Stefano, Vincenzo Brescia Morra, Alvino Bisecco, Giancarlo Coghe, Eleonora Cocco, Michele Romoli, Francesco Corea, Letizia Leocani, Jessica Frau, Simona Sacco, Matilde Inglese, Antonio Carotenuto, Roberta Lanzillo, Alessandro Padovani, Maria Triassi, Simona Bonavita, Luigi Lavorgna, Digital Technologies, Web, Social Media Study Group of the Italian Society of Neurology (SIN)

**Affiliations:** 1 Department of Advanced Medical and Surgical Sciences, University of Campania “Luigi Vanvitelli”, Via Pansini 5, 80131 Naples, Italy; 2 Department of Molecular Medicine and Medical Biotechnology, Federico II University of Naples, Naples, Italy; 3 Multiple Sclerosis Unit, Policlinico Federico II University Hospital, Naples, Italy; 4 Department of Public Health, University “Federico II” of Naples, Naples, Italy; 5 Department of Primary Care and Public Health, Imperial College of London, London, UK; 6 Department of Human Neuroscience, Sapienza University of Rome, Rome, Italy; 7 Dipartimento di Scienze Cliniche e Biologiche, Università Di Torino, Turin, Italy; 8 Department of Neurology 2 and Tuscan Region Multiple Sclerosis Referral Centre, Careggi University Hospital, Florence, Italy; 9 Department of Neurology and Multiple Sclerosis Regional Referral Centre, AOU San Luigi Gonzaga, Orbassano, Turin, Italy; 10 Department of Neurosciences, Reproductive Sciences and Odontostomatology, University of Naples Federico II, Naples, Italy; 11 Department of Neuroscience, Azienda Ospedaliero-Universitaria di Modena, Modena, Emilia-Romagna Italy; 12 Department of Biomedical, Metabolic and Neural Sciences, University of Modena and Reggio Emilia, Modena, Italy; 13 Department of Medicine, Surgery and Neuroscience, University of Siena, Siena, Italy; 14 Multiple Sclerosis Center, Department of Advanced Medical and Surgical Sciences, University of Campania “Luigi Vanvitelli”, Naples, Italy; 15 Institute of Neurology, University Magna Graecia of Catanzaro, Catanzaro, Italy; 16 Neurology Unit, Bufalini Hospital, Local Health Agency of Romagna, Cesena, Italy; 17 Department of Brain Sciences, Imperial College London, London, W120BZ UK; 18 Neurology Unit-Neuroscience Department A.S.S.T.Lecco, Merate Hospital, 23807 Merate, Italy; 19 Neurology Unit, Fondazione IRCCS Casa Sollievo della Sofferenza, 71013 San Giovanni Rotondo, Italy; 20 Department of Neuroscience, Rehabilitation, Ophthalmology, Genetics, Maternal and Child Health (DiNOGMI), University of Genoa, Genoa, Italy; 21 Department of Neurology, IRCSS Ospedale Policlinico San Martino, Genoa, Italy; 22 Department of Biotechnological and Applied Clinical Sciences, University of L’Aquila, L’Aquila, Italy; 23 Neurology Unit, Multiple Sclerosis Center, Fabrizio Spaziani Hospital, Frosinone, Italy; 24 Department of Neurology and Stroke Unit of Avezzano-Sulmona, ASL 1 Avezzano-Sulmona-L’Aquila, L’Aquila, Italy; 25 Department of Neuroscience, Multiple Sclerosis Center, S. Maria delle Croci Hospital, AUSL Romagna, Ravenna, Italy; 26 Department of Medical Sciences and Public Health, Multiple Sclerosis Center, Binaghi Hospital, ASL Cagliari, University of Cagliari, Cagliari, Italy; 27 Dipartimento di Neurologia, Ospedale di Foligno, Azienda USL Umbria 2, Terni, Italy; 28 Vita-Salute San Raffaele University, Milan, Italy; 29 Experimental Neurophysiology Unit, Institute of Experimental Neurology-INSPE, IRCCS Scientific Institute San Raffaele, Milan, Italy; 30 Unit of Neurology, Azienda Socio-Sanitaria Territoriale Spedali Civili, Brescia, Italy; 31 Department of Clinical and Experimental Sciences, University of Brescia, Brescia, Italy

**Keywords:** Artificial intelligence, Machine learning, Multiple sclerosis, Large language model

## Abstract

**Background:**

ChatGPT is an open-source natural language processing software that replies to users’ queries. We conducted a cross-sectional study to assess people living with Multiple Sclerosis’ (PwMS) preferences, satisfaction, and empathy toward two alternate responses to four frequently-asked questions, one authored by a group of neurologists, the other by ChatGPT.

**Methods:**

An online form was sent through digital communication platforms. PwMS were blind to the author of each response and were asked to express their preference for each alternate response to the four questions. The overall satisfaction was assessed using a Likert scale (1–5); the Consultation and Relational Empathy scale was employed to assess perceived empathy.

**Results:**

We included 1133 PwMS (age, 45.26 ± 11.50 years; females, 68.49%). ChatGPT’s responses showed significantly higher empathy scores (Coeff = 1.38; 95% CI = 0.65, 2.11; *p* > *z* < 0.01), when compared with neurologists’ responses. No association was found between ChatGPT’ responses and mean satisfaction (Coeff = 0.03; 95% CI = − 0.01, 0.07; *p* = 0.157). College graduate, when compared with high school education responder, had significantly lower likelihood to prefer ChatGPT response (IRR = 0.87; 95% CI = 0.79, 0.95; *p* < 0.01).

**Conclusions:**

ChatGPT-authored responses provided higher empathy than neurologists. Although AI holds potential, physicians should prepare to interact with increasingly digitized patients and guide them on responsible AI use. Future development should consider tailoring AIs’ responses to individual characteristics. Within the progressive digitalization of the population, ChatGPT could emerge as a helpful support in healthcare management rather than an alternative.

**Supplementary Information:**

The online version contains supplementary material available at 10.1007/s00415-024-12328-x.

## Introduction

Clinical practice is quickly changing following digital and technological advances, including artificial intelligence (AI) [1–4]. ChatGPT [5] is an open-source generative processing AI software that proficiently replies to users’ queries, in a human-like modality [6]. Since its first iteration (November 2022), ChatGPT's popularity has been growing exponentially. In its most recent release (January 2023), the number of users exceeded the threshold of 100,000 [7]. ChatGPT was not specifically developed to provide healthcare opinion, but replies to a wide range of questions, including those health-related [8]. Thus, ChatGPT could represent an easily-accessible resource to seek health information and advice [9, 10].

The search for health information is particularly relevant in people living with chronic diseases [11], inevitably facing innumerable challenges, including communication with their healthcare providers. We decided to focus on people living with Multiple Sclerosis (PwMS), as a model of chronic disease that can provide insights applicable to the broader landscape of chronic diseases. The young age of onset of MS results in high patients’ digitalization, including the use of mobile health apps, remote monitoring devices, and AI-based tools [12]. The increasing engagement by patients with AI platforms to ask questions related to their MS is a reality that clinicians will probably need to confront.

We conducted a comparative analysis to investigate the perspective of PwMS towards two alternate responses to four frequently-asked health-related questions. The responses were authored by a group of neurologists and by ChatGPT, with PwMS unaware of whether they were formulated by neurologists or generated by ChatGPT. The aim was to assess patients’ preferences, overall satisfaction, and perceived empathy between the two options.

## Methods

### Study design and form preparation

This is an Italian multicenter cross-sectional study, conducted from 09/01/2023 to 09/20/2023. The study conduction and data presentation followed the Strengthening the Reporting of Observational Studies in Epidemiology (STROBE) statements [13].

The study was conducted within the activities of the “Digital Technology, Web and Social Media” study group [14], which includes 205 neurologists, affiliated with the Italian Society of Neurology (SIN). The study invitation was disseminated to all members of the group via the official mailing list.

Following the invitation to participate, neurologists were required to meet the following criteria:

i. Dedicate over 50% of their clinical time to MS care and be active outpatient practitioners;
ii. Regularly receive and respond to patient-generated emails or engage with patients on web platform or other social media.

Only the 34 neurologists who met the specified criteria were included and, using Research Randomizer [15], were randomly assigned to four groups. Demographic information is presented in Table 1 of Online Resources.

- Group A, including 5 neurologists, was required to identify a list of frequently-asked questions based on the actual queries of PwMS received via e-mail in the preceding 2 months. The final list, drafted by Group A, was composed of fourteen questions.
- Group B, including 19 neurologists, had to identify the four questions they deemed the most common and relevant for clinical practice (from the fourteen elaborated by Group A). Group B was deliberately designed as the largest group to ensure a more comprehensive and representative selection of the questions. The four identified questions were 1. “I feel more tired during summer season, what shall I do?"; 2. “I have had new brain MRI, and there is one new lesion. What should I do?"; 3. “Recently, I’ve been feeling tired more easily when walking long distances. Am I relapsing?”; 4. “My primary care physician has given me a prescription for an antibiotic for a urinary infection. Is there any contraindication for me?”.
- Group C, including 5 neurologists, focused on elaborating the responses to the four questions identified as the most common by Group B. The responses were collaboratively formulated through online meetings. Any discrepancies were addressed through discussion and consensus.
  Afterwards, the same questions were submitted to ChatGPT 3.5, which provided its own version of the answers. Hereby:
- Group D, including 5 neurologists, carefully reviewed the responses generated by ChatGPT to identify any inaccuracies in medical information or discrepancies from the recommendations before submitting to PwMS (none were identified and, thus, no changes were required).

Questions and answers are presented in the full version of the form in Online Resources.

Subsequentially, we designed an online form to explore the perspective of PwMS on the two alternate responses to the four common questions, those authored by Group C neurologists and the others by the open-source AI tool (ChatGPT). PwMS were unaware of whether the responses were formulated by neurologists or generated by ChatGPT. The workflow process is illustrated in Fig. 1 in Online Resources.

The study was conducted in accordance with the guidelines of the Declaration of Helsinki involving human subjects and the patient’s informed consent was obtained at the outset of the survey. The Ethical Committee of the University of Campania “Luigi Vanvitelli” approved the study (protocol number 0014460/i).

### MS population and variables

PwMS were invited to participate to the study by their neurologists, through different communication tools, such as institutional e-mail and instant messaging platform. A total of 2854 invites were sent from 09/01/2023 to 09/20/2023.

The study covariates included demographic information (year of birth, sex, area of residence in Italy, and level of education, defined as elementary school, middle school, high school graduate or college graduate, the latter encompassing both a bachelor's degree and post-secondary education) and clinical characteristics (depressed mood, subjective memory and attention deficits, year of MS diagnosis, MS clinical descriptors, such as relapsing–remitting—RRMS, secondary-progressive—SPMS, primary-progressive—PPMS, or “I don’t know”). The occurrence of depressive mood was surveyed using the Patient Health Questionnaire (PHQ)-2 scale [16]. The rationale behind the decision to employ this rating scale was the rapidity of completion and its widespread use in the previous online studies including PwMS [17, 18]. Subjective memory and attention impairment deficits were investigated by directly asking patients about their experience on these symptoms (yes/no).

Preference between alternate responses was investigated by asking patients to express their choice. Furthermore, for each response, a Likert scale ranging from 1 to 5 was provided to assess overall satisfaction (higher scores indicating higher satisfaction). The Consultation and Relational Empathy (CARE) scale [19] was employed to evaluate the perceived empathy of the different responses (higher scores indicating higher empathy). The CARE scale measures empathy within the context of the doctor-patient relationship, and was ultimately selected for its intuitiveness, easiness of completion, and because already used in online studies [20, 21]. Given the digital nature of our research, we made a single wording adjustment to the CARE scale to better align to our study. Further details on the form (Italian original version and English translated version) and measurement scales are presented in Online Resources.

Prior to submitting the form to the patients, overall readability level was assessed. All responses elaborated by the neurologists and by ChatGPT were analysed by two validated tools for the Italian language: the Gulpease index [22] and the Read-IT scores (version 2.1.9) [23]. This step was deemed meaningful for a thorough and comprehensive appraise of all possible factors that could influence patients' perceptions.

### Statistical analysis

The study variables were described as mean (standard deviation), median (range), or number (percent), as appropriate.

The likelihood of selecting the ChatGPT response for each question was evaluated through a stratified analysis employing logistic regression models; this approach was adopted to address variations in the nature of the questions. The selection rate of answers generated by ChatGPT was assessed using Poisson regression models. The continuous outcomes (average satisfaction and average CARE scale scores for ChatGPT responses) were assessed using mixed linear models with robust standard errors accounting for heteroskedasticity across patients. Covariates were age, sex, treatment duration, clinical descriptors, presence of self-reported cognitive deficit, presence of depressive symptoms and educational attainment. The software consistently selected the first level of the categorical variable, alphabetically or numerically, as the reference group to ensure straightforward interpretation of coefficients or effects.

The results were reported as adjusted coefficient (Coeff), odds ratio (OR), incidence rate ratio (IRR), 95% confidence intervals (95% CI), and *p* values, as appropriate. The results were considered statistically significant for *p* < 0.05. Statistical analyses were performed using Stata 17.0.

## Results

The study included 1133 PwMS (age, 45.26 $\pm$ 11.50 years; females, 68.49%), with an average response rate of 39.70%. Demographic and clinic characteristics are summarized in Table 1.

**Table 1:** Demographic and clinical characteristics of the study population (*N* = 1133)

Age, mean (SD), years	45.26 (11.50)
Female, No (%)	776 (68.49)
Education, No (%)	
Elementary school	7 (0.62)
Middle school	134 (11.83)
High school	575 (50.75%)
College graduates	417 (36.80%)
Geographical origin, No (%)	
Northern-Italy	247 (21.80)
Centre-Italy	527 (46.51)
Southern-Italy	282 (24.89)
Island	77 (6.80)
Duration of MS disease, mean (SD), years	12.28 (9.61)
MS clinical descriptors, No (%)	
RRMS	800 (70.61)
SPMS	100 (8.83)
PPMS	61 (5.38)
“I don’t know”	172 (15.18)

*MS* Multiple sclerosis, *n*: number, *PPMS* primary progressive multiple sclerosis, *RRMS* relapsing–remitting multiple sclerosis, *SD* standard deviation, *SPMS* secondary-progressive multiple sclerosis

Table 2 provides an overview of participant preferences, mean satisfaction (rated on a Likert scale ranging from 1 to 5), and CARE scale scores for each response by ChatGPT and neurologists to the four questions.

**Table 2:** Participant preferences, mean satisfaction (rated on a Likert scale ranging from 1 to 5), and CARE scale mean scores for each response

	ChatGPT	Neurologist
Question 1		
Preference, *n* (%)	779 (68.76%)	354 (31.24%)
Likert scale (1–5), mean (SD)	3.63 (1.03)	3.02 (1.19)
CARE scale, mean (SD)	34.24 (8.09)	28.91 (9.47)
Question 2		
Preference, *n* (%)	672 (59.31%)	461 (40.69%)
Likert scale (1–5), mean (SD)	3.62 (1.22)	3.30 (1.09)
CARE scale, mean (SD)	31.25 (9.71)	30.89 (8.53)
Question 3		
Preference, *n* (%)	437 (38.57%)	696 (61.43%)
Likert scale (1–5), mean (SD)	3.22 (1.08)	3.63 (1.19)
CARE scale, mean (SD)	32.38 (9.02)	31.58 (9.51)
Question 4		
Preference, *n* (%)	430 (37.95%)	703 (62.05%)
Likert scale (1–5), mean (SD)	3.17 (1.24)	3.57 (1.04)
CARE scale, mean (SD)	29.54 (9.74)	30.87 (8.61)

*n* Number, *SD* standard deviation

Univariate analyses did not show significant differences in preferences. However, after adjusting for factors potentially influencing the outcome, emerged that the likelihood of selecting ChatGPT response was lower for college graduates when compared with respondent with high school education (IRR = 0.87; 95% CI = 0.79, 0.95; *p* < 0.01).

Further analysis of each singular question resulted in additional findings summarized in Table 3.

**Table 3:** Multivariate logistic regressions

	All questions		Question 1		Question 2		Question 3		Question 4	
	IRR (95% CI)	*p*-value	OR (95% CI)	*p-*value	OR (95% CI)	*p-*value	OR (95% CI)	*p-*value	OR (95% CI)	*p-*value
Age, years	0.99 (0.99–1.00)	0.381	0.98 (0.97–0.99)	**0.021**	1.00 (0.99–1.02)	0.273	0.98 (0.97–1.00)	0.066	1.00 (0.98–1.01)	0.796
Sex (Ref. other)										
Female	1.48 (0.68–3.19)	0.316	3.57 (0.50–25.60)	0.204	0.59 (0.06–5.83)	0.659	2.31 (0.20–26.06)	0.497	0.92 (0.70–1.21)	0.575
Male	1.53 (0.71–3.31)	0.277	4.23 (0.58–30.47)	0.152	0.55 (0.05–5.46)	0.617	2.73 (0.24–31.03)	0.416	0.82 (0.81–1.35)	0.574
Education (Ref. high school graduate)										
Elementary school	0.85 (0.49–1.48)	0.576	1.46 (0.26–8.15)	0.662	0.29 (0.06–1.39)	0.124	2.64 (0.54–12.85)	0.229	0.17 (0.02–1.47)	0.108
Middle school	1.02 (0.90–1.17)	0.675	1.16 (0.74–1.79)	0.506	0.84 (0.56–1.25)	0.398	1.19 (0.80–1.76)	0.379	1.11 (0.75–1.66)	0.087
College graduates	0.87 (0.79–0.95)	** < 0.01**	0.64 (0.48–0.85)	** < 0.01**	0.72 (0.55–0.95)	**0.019**	0.80 (0.61–1.05)	0.108	0.78 (0.59–1.03)	0.584
Duration of MS disease, years	1.00 (0.99–1.00)	0.711	1.00 (0.98–1.01)	0.736	0.99 (0.98–1.01)	0.711	1.01 (0.99–1.02)	0.066	0.99 (0.98–1.01)	0.502
MS clinical descriptors (Ref. “PPMS”)										
RRMS	0.86 (0.72–1.03)	0.114	0.45 (0.22–0.90)	**0.025**	0.66 (0.37–1.19)	0.169	0.78 (0.44–1.37)	0.460	0.93 (0.52–1.64)	0.804
SPMS	0.90 (0.72–1.13)	0.383	0.36 (0.16–0.80)	**0.012**	0.83 (0.41–1.68)	0.610	0.47 (0.23–0.96)	**0.038**	2.19 (1.10–4.35)	**0.025**
“I don’t know”	0.91 (0.74–1.11)	0.365	0.44 (0.20–0.93)	**0.032**	0.78 (0.41–1.48)	0.451	0.79 (0.42–1.47)	0.460	1.31 (0.70–2.47)	0.393
Depression	0.94 (0.86–1.02)	0.173	0.67 (0.51–0.89)	** < 0.01**	1.08 (0.84–1.40)	0.526	0.81 (0.63–1.05)	0.127	0.93 (0.71–1.27)	0.624
Subjective memory and attention deficit	1.03 (0.94–1.13)	0.423	0.79 (0.56–1.04)	0.097	1.39 (1.06–1.81)	**0.014**	0.84 (0.64–1.09)	0.203	1.45 (1.11–1.90)	** < 0.01**

The main outcome (likelihood of selecting the ChatGPT response) was evaluated through a stratified analysis employing logistic regression models, by assessing the frequency of selecting the ChatGPT option divided by the total number of questions. This approach was adopted to address variations in the nature of the questions. Covariates were age, sex, treatment duration, clinical descriptors, self-reported cognitive deficit, depressive symptoms and educational attainment

*CI* Confidence interval, *IRR* incidence rate ratio, *MS* multiple sclerosis, *OR* odds ratio, *PPMS* primary progressive multiple sclerosis, *RRMS* relapsing–remitting multiple sclerosis, *SPMS* secondary-progressive multiple sclerosis; the use of bold formatting within the table was employed to highlight significant values

Although there was no association between the ChatGPT responses and satisfaction (Coeff = 0.03; 95% CI = − 0.01, 0.07; *p* = 0.157), they exhibited higher CARE scale scores (Coeff = 1.38; 95% CI = 0.65, 2.11; *p* > *z* < 0.01), as compared to the responses processed by neurologists. The findings are summarized in Table 4.

**Table 4:** Mixed linear regressions

	Satisfaction		CARE scale	
	Coeff (95% CI)	*p* value	Coeff (95% CI)	*p* value
ChatGPT (Ref. neurologists)	0.03 (− 0.01 to 0.07)	0.157	1.37 (0.64 to 2.11)	** < 0.01**
Age, years	0.00 (− 0.00 to 0.00)	0.441	0.00 (− 0.03 to 0.04)	0.867
Sex				
Female	− 0.33 (− 0.91 to 0.25)	0.265	− 5.56 (− 11.61 to 0.48)	0.071
Male	− 0.35 (− 0.93 to 0.23)	0.241	− 6.05 (− 12.11 to 0.00)	**0.050**
Education (Ref. high school graduate)				
Elementary school	− 0.63 (− 1.11 to − 0.15)	**0.01**	− 2.24 (− 7.21 to 2.72)	0.376
Middle school	0.021 (− 0.10 to 0.14)	0.732	2.27 (0.494 to 0.05)	0.369
College graduates	− 0.007 (− 0.089 to 0.074)	0.857	− 1.08 (− 1.93 to − 0.24)	**0.012**
Duration of MS disease, years	− 0.00 (− 0.00 to 0.001)	0.251	− 0.02 (− 0.07 to 0.01)	0.206
MS clinical descriptors (Ref. PPMS)				
RRMS	0.19 (0.00 to 0.38)	**0.024**	2.27 (0.49 to 4.05)	**0.012**
SPMS	0.14 (0.02 to 0.36)	0.171	1.83 (− 0.32 to 3.99)	0.097
“I don’t know”	0.19 (− 0.06 to 0.35)	**0.048**	1.90 (− 0.06 to 3.87)	0.058
Depression	0.04 (− 0.04 to 0.11)	0.355	− 0.68 (− 1.50 to 0.12)	0.098
Subjective memory and attention deficit	0.03 (− 0.05 to 0.11)	0.431	0.19 (− 0.63 to 1.03)	0.641

The continuous outcomes (average satisfaction and average CARE scale scores for ChatGPT responses) were assessed using mixed linear models with robust standard errors accounting for heteroskedasticity across patients. Covariates were age, sex, treatment duration, clinical descriptors, presence of self-reported cognitive deficit, presence of depressive symptoms and educational attainment

*CI* Confidence interval, *IRR* incidence rate ratio, *MS* multiple sclerosis, *OR* odds ratio, *PPMS* primary progressive multiple sclerosis, *RRMS* relapsing–remitting multiple sclerosis, *SPMS* secondary-progressive multiple sclerosis; *y* years; the use of bold formatting within the table was employed to highlight significant values

The readability of the answers provided by ChatGPT and neurologists was medium, as assessed with the Gulpease Index. Although similar, Gulpease indices were slightly higher for ChatGPT’s responses than for neurologists (ChatGPT: from 47 to 52; neurologists: from 40 to 44). The results were corroborated by ReadIT scores, which are inversely correlated with Gulpease Index. Table 5 shows the readability of each response.

**Table 5:** Mean readability indices for each question

Readability index		Question 1	Question 2	Question 3	Question 4	Minimum–maximum—ChatGPT	Minimum–maxmum—neurologist
Gulpease index	ChatGPT	52	49	48	47	47–52	40–44
	Neurologist	40	44	42	40		
ReadIt—base	ChatGPT	32.3%	55.5%	56.7%	65.9%	32.3–65.9	88.9–98.7
	Neurologist	96.6%	88.9%	98.7%	98.0%		
ReadIt—lexical	ChatGPT	74.4%	24.9%	96.0%	70.4%	24.9–96	80.2–100
	Neurologist	100%	89.3%	93.3%	80.2%		
ReadIt—syntax	ChatGPT	21.7%	36.3%	95.7%	92.1%	21.7–95.7	15.1–99.6
	Neurologist	78.3%	15.1%	99.6%	95.5%		
ReadIt—global	ChatGPT	2.0%	0.8%	71.9%	50.8%	0.8–71.9	90.7–100
	Neurologist	100%	90.7%	100%	100%		

The Gulpease index evaluates the overall readability of a text on a scale of 0 to 100, with higher scores indicating better ease of reading. A Gulpease index between 40 and 60 denotes a text that is poorly understandable to individuals with an elementary or middle school license, but easily comprehensible to high school graduates and those with higher education. Conversely, the Read-IT scores assess the different layers of a text, namely the structural, syntactic, and lexical plan, thereby rendering four distinct indices, Read-IT Base, Read-IT Lexical, Read-IT Syntax and Read-IT Global

## Discussion

The Internet and other digital tools, such as AI, have become a valuable source of health information [24, 25]. Seeking answers online requires minimal effort and guarantees immediate results, making it more convenient and faster than contacting healthcare providers. AIs, like ChatGPT can be viewed as a new, well-structured search engine with a simplified, intuitive interface. This allows patients to submit questions and receive direct answers, eliminating the need to navigate multiple websites [26]. However, there is the risk that internet and AI-based health information provides incomplete or incorrect information, along with potentially reduced empathy of communication [27–29]. Our study examined participant preferences, satisfaction ratings and perceived empathy regarding responses generated by ChatGPT as compared to those from neurologists. Interestingly, although ChatGPT responses did not significantly affect satisfaction levels, they were perceived as more empathetic compared to responses from neurologists. Furthermore, after adjusting for confounding factors, including education level, our results revealed that college graduates showed less inclination to choose ChatGPT responses compared to individuals with a high school education. This highlights how individual preferences are not deterministic but could instead be influenced by a variety of factors, including age, education level, and others [30, 31].

In line with the previous study [32], ChatGPT provided sensible responses, which were deemed more empathetic than those authored by neurologists. A plausible explanation for this outcome may lie in the observation that ChatGPT's responses showed a more informal tone^1^ when addressing patients’ queries. Furthermore, ChatGPT tended to include empathetic remarks and language implying solidarity (i.e., a welcoming remark of gratitude and a sincere-sounding invitation for further communication). Thus, PwMS, especially those with lower level of education, might perceive confidentiality and informality as empathy. Moreover, the lower empathy shown by neurologists could be related to job well-being factors, including feelings of overwhelming and work overload (i.e., allocation of time to respond to patients’ queries). Even though our study did not aim to identify the reasons behind participants' ratings, these findings might represent a potential direction for future research.

Another relevant finding was that PwMS with higher levels of education showed lower satisfaction towards the responses developed by ChatGPT, this suggest that educational level could be a key factor in health communication. Several studies suggest that having a higher degree of education is associated with a better predisposition towards AIs [33], and in general, towards online information seeking [34, 35]. Although it may seem a contradiction, the predisposition toward digital technology doesn’t necessarily align with the perception of communicative messages within the doctor-patient relationship. Moreover, people with higher levels of education may have developed greater critical skills, enabling them to better appreciate the appropriateness and precision of the language employed by neurologists [30].

In addition, in our study, the responses provided by ChatGPT have shown adequate overall readability, using simple words and clear language. This could be one of the reasons that make them potentially more favourable for individuals with lower levels of education and for younger people.

When examining individually the four questions, we observed varying results without a consistent pattern; however, no contradictory findings emerged.

In questions N° 2 and N° 4, PwMS who reported subjective memory and attention deficits were more likely to select the AI response. Still, in question N° 1 and N° 4, a higher likelihood to prefer the ChatGPT response emerged for subjects with PPMS. This result could be attributed to the distinct cognitive profile showed in PPMS [36], which is characterized by moderate-to-severe impairments.

In addition, for question N° 1, there was a decrease in the probability of preferring the response generated by ChatGPT with the increasing age of the participants. This result is in line with some previous findings [34, 35], and further highlights the digital divide between “Digital Natives,” those who were born into digital age, and “Digital Immigrants”, those who experienced the transition to digital [37, 38].

Finally, in question N° 1, PwMS with depressive symptoms showed a lower propensity to select the response generated by ChatGPT. This result could suggest that ChatGPT employs a type of language and vocabulary that is perhaps less well-received by individuals with depressive symptoms than the one used by neurologists, leading them to prefer the latter. Further research with a combination of quantitative and qualitative tools is needed to deepen this insight.

Our results point to the need to tailor digital resources, including ChatGPT, to render them more accessible and user-friendly for all users, considering their needs and skills. This could help bridge the present gap and enable digital resources to be effective for a wide range of users, regardless of their age, education, and medical and digital background. Indeed, a significant issue is that ChatGPT lacks knowledge of the details and background data of PwMS medical record, as it could lead to inaccurate or incorrect advice.

Furthermore, as our findings showed greater empathy of ChatGPT towards PwMS queries, the concern is that they may over-rely on AI rather than consulting their neurologist. Given the potential risks associated with the unsupervised use of AIs, physicians are encouraged to adapt to the progressive digitization of patients. This includes not only providing proper guidance on the use of digital resources for health information seeking, but also addressing the potential drawbacks associated with relying solely on AI-generated results. Moreover, future research should address the possible integration of chatbots into a mixed-mode framework (AI-assisted approach). Integrating generative AI software into the neurologist's clinical practice could facilitate efficient communication while maintaining the human element.

Our study has several strengths and limitations. The objective was to explore the potential of AIs, such as ChatGPT, in the interaction with PwMS by engaging patients themselves in the evaluation [9, 10]. To this aim, we have replicated a patient-neurologist or patient-AIs interaction scenario. ChatGPT open access version 3.5 was preferred over newer and more advanced version 4.0 (pay per use). In fact, nonmajor users of online services will likely seek information free of charge [39, 40].

The main limitation of our study is the relatively small number of questions, however we deliberately selected a representative sample to enhance compliance and avoid discouraging PwMS from responding [41], as length can be a factor affecting response rates. Moreover, while our study adopted MS as a model of chronic disease for its core features, findings are not generalizable to other chronic conditions. We employed subjective self-report of cognitive impairment, and more studies adopting objective measures of cognitive screening will be needed to confirm our findings. Because a high level of education does not always correspond to a high digital literacy, it will be essential to assess digital literacy in future studies. Moreover, we preferred the use of institutional e-mail and instant messaging platform for recruitment, over in-person participation, given the predominantly digital nature of the research; still, the average response rate was in line with previous research [42]. We acknowledged that using stratified models may entail the risk of incurring Type I errors. However, stratification has been applied within homogeneous subgroups and on outcomes that are contextually differentiated due to the nature of different questions. This targeted approach allows for more accurate associations between predictors and outcomes to be discovered, thereby minimizing the risk of Type I error. Given the nonuniform distribution of patients across education classes within the categorical variable and the direct relationship between statistical significance and sample size, it's conceivable that this influenced the outcomes for the lower education classes (elementary and high school). However, the already observed statistically significant difference among the higher education classes implies that similar results could be achieved by standardizing the distribution of patients within the "education" categorical variable. This highlights the potential significance of education level in health communication, a crucial aspect warranting further exploration in scientific research. Finally, we tested ChatGPT in the perceived quality of communication, and did not assess its ability to make actual clinical decisions, which would require further specific studies.

Future development should include to (a) guide the development of AI-based systems that better meet the needs and preferences of patients, taking into consideration their cultural, social and digital backgrounds; (b) educate healthcare professionals and patients on AI's role and capabilities for an informed and responsible use; (c) implement research methodology in the field of remote healthcare communication.

## Conclusion

Our study showed that PwMS find ChatGPT's responses more empathetic than those of neurologists. However, it seems that ChatGPT is not completely ready to fully meet the needs of some categories of patients (i.e., high educational attainment). While physicians should prepare themselves to interact with increasingly digitized patients, ChatGPT’s algorithms needs to focus on tailoring its responses to individual characteristics. Therefore, we believe that AI tools may pave the way for new perspectives in chronic disease management, serving as valuable support elements rather than alternatives.

### Supplementary Information

Below is the link to the electronic supplementary material.

Supplementary file1 (DOCX 613 KB)

## Data Availability

The data that support the findings of this study are available from the corresponding author, SB, upon reasonable request.

## Footnotes

1 For clarification, in Italian grammar, the use of the second or third person is based on levels of formality and familiarity. While the second person singular is more informal, the third person singular is employed in more formal or professional contexts, reflecting a respectful or polite tone.
