# Supplementary material for: ChatGPT vs. neurologists: a cross-sectional study investigating preference, satisfaction ratings and perceived empathy in responses among people living with multiple sclerosis
Source: J Neurol. 2024 Apr 3;271(7):4057–66. doi: 10.1007/s00415-024-12328-x (PMC11233331; doi:10.1007/s00415-024-12328-x)
Supplement: Supplementary file 1 — Supplementary file1 (DOCX 613 KB) [file 415_2024_12328_MOESM1_ESM.docx]

**ChatGPT vs. Neurologists: a Cross-Sectional Study Investigating Preference, Satisfaction Ratings and Perceived Empathy in Responses among People Living with Multiple Sclerosis.**

**Journal of Neurology**

Elisabetta Maida^1#^, Marcello Moccia^2,3#^, Raffaele Palladino^4,5^, Giovanna Borriello^6^, Giuseppina Affinito^4^, Marinella Clerico^7^, Anna Maria Repice^8^, Alessia Di Sapio^9^, Rosa Iodice^10^, Antonio Luca Spiezia^3^, Maddalena Sparaco^1^, Giuseppina Miele^1^, Floriana Bile^1^, Cristiano Scandurra^10^, Diana Ferraro^11,12^, Maria Laura Stromillo^13^, Renato Docimo^14^, Antonio De Martino^15^, Luca Mancinelli^16^, Gianmarco Abbadessa^1,17^, Krzysztof Smolik^12^, Lorenzo Lorusso^18^, Maurizio Leone^19^, Elisa Leveraro^20,21^, Francesca Lauro^10^, Francesca Trojsi^1^, Lidia Mislin Streito^7^, Francesca Gabriele^22^, Fabiana Marinelli^23^, Antonio Ianniello^6^, Federica De Santis^24^, Matteo Foschi^22,25^, Nicola De Stefano^13^, Vincenzo Brescia Morra^10^, Alvino Bisecco^14^, Giancarlo Coghe^26^, Eleonora Cocco^26^, Michele Romoli^16^, Francesco Corea^27^, Letizia Leocani^28,29^, Jessica Frau^26^, Simona Sacco^22^, Matilde Inglese^20,21^, Antonio Carotenuto^10^, Roberta Lanzillo^10^, Alessandro Padovani^30,31^, Maria Triassi^4^, Simona Bonavita^1^*, Luigi Lavorgna^1^, *on behalf of Digital Technologies, Web, Social Media Study Group of the Italian Society of Neurology (SIN)*

*^1^ Department of Advanced Medical and Surgical Sciences, University of Campania “Luigi Vanvitelli”, Naples, Italy*

*^2^ Department of Molecular Medicine and Medical Biotechnology, Federico II University of Naples, Italy*

*^3^ Multiple Sclerosis Unit, Policlinico Federico II University Hospital, Naples, Italy*

*^4^ Department of Public Health, University "Federico II" of Naples, Italy*

*^5^ Department of Primary Care and Public Health, Imperial College of London, the UK*

*^6^ Department of human Neuroscience, Sapienza University of Rome, Rome, Italy*

*^7^ Dipartimento di Scienze Cliniche e Biologiche, Università di Torino, Turin, Italy*

*^8^ Department of Neurology 2 and Tuscan Region Multiple Sclerosis Referral Centre, Careggi University Hospital, Florence, Italy*

*^9^ Department of Neurology and Multiple Sclerosis Regional Referral Centre, AOU San Luigi Gonzaga, Orbassano, Torino, Italy*

*^10^ Department of Neurosciences, Reproductive Sciences and Odontostomatology, University of Naples Federico II, Napoli, Italy*

*^11^ Department of Neuroscience, Azienda Ospedaliero-Universitaria di Modena, Modena, Emilia-Romagna, Italy*

*^12^ Department of Biomedical, Metabolic and Neural Sciences, University of Modena and Reggio Emilia, Modena, Italy*

*^13^ Dept. of Medicine, Surgery and Neuroscience, University of Siena, Siena, Italy.*

*^14^ Multiple Sclerosis Center, Dept. of Advanced Medical and Surgical Sciences, University of Campania "Luigi Vanvitelli", Naples, Italy*

*^15^ Institute of Neurology, University Magna Graecia of Catanzaro, Italy*

*^16^ Neurology Unit, Bufalini Hospital, Local Health Agency of Romagna, Cesena, Italy*

*^17^* *Department of Brain Sciences, Imperial College London, London W120BZ, UK*

*^18^ Neurology Unit-Neuroscience Dept. A.S.S.T.Lecco, Merate Hospital, 23807 Merate, Italy*

*^19^ Neurology Unit, Fondazione IRCCS Casa Sollievo della Sofferenza, 71013 San Giovanni Rotondo, Italy*

*^20^ Department of Neuroscience, Rehabilitation, Ophthalmology, Genetics, Maternal and Child Health (DiNOGMI), University of Genoa, Genoa, Italy*

*^21^ IRCSS Ospedale Policlinico San Martino, Department of Neurology, Genova, Italy*

*^22^ Department of Biotechnological and Applied Clinical Sciences, University of L'Aquila, L'Aquila, Italy*

*^23^ Neurology Unit, Multiple Sclerosis Center, Fabrizio Spaziani Hospital, Frosinone, Italy*

*^24^ Department of Neurology and Stroke Unit of Avezzano-Sulmona, ASL 1 Avezzano-Sulmona-L’Aquila, L’Aquila, Italy*

*^25^ Department of Neuroscience, Multiple sclerosis Center, S.Maria delle Croci Hospital, AUSL Romagna, Ravenna, Italy*

*^26^ Department of Medical Sciences and Public Health, Multiple Sclerosis Center, Binaghi Hospital, ASL Cagliari, University of Cagliari, Cagliari, Italy*

*^27^ Dipartimento di Neurologia, Ospedale di Foligno, Azienda USL Umbria 2*

*^28^ Vita-Salute San Raffaele University, Milan, Italy*

*^29^ IRCCS Scientific Institute San Raffaele, Experimental Neurophysiology Unit, Institute of Experimental Neurology-INSPE, Milan, Italy*

*^30^ Unit of Neurology, Azienda Socio-Sanitaria Territoriale Spedali Civili, Brescia, Italy*

*^31^ Department of Clinical and Experimental Sciences, University of Brescia, Brescia, Italy*

*^#^These authors contributed equally to this work as cofirst authors.*

***Corresponding author:**

**Simona Bonavita**

Department of Advanced Medical and Surgical Sciences, University of Campania Luigi Vanvitelli, Via Pansini 5, 80131, Naples, Italy

Tel: +39 081 566 6742; e-mail: [simona.bonavita@unicampania.it](mailto:simona.bonavita@unicampania.it)

**Full version of the form (original Italian version)**

*Per maggiore chiarezza, in questa versione tradotta abbiamo indicato con un asterisco le risposte fornite da ChatGPT da sottoporre all'attenzione del revisore.*

*Le quattro domande sono state presentate sia ai neurologi che a ChatGPT con la seguente traccia: "Come risponderesti a un paziente con sclerosi multipla che ti chiede..."*

Demografica

1. Sesso

- M
- F
- Altro
- Preferisco non dire

1. Anno di nascita
2. In che regione vive

- Abbruzzo
- Basilicata
- Calabria
- Campania
- Emilia-Romagna
- Friuli-Venezia Giulia
- Lazio
- Liguria
- Lombardia
- Marche
- Molise
- Piemonte
- Puglia
- Sardegna
- Sicilia
- Toscana
- Trentino-Alto Adige
- Umbria
- Valle d’Aosta
- Veneto

1. Che scuole ha completato

- Elementari
- Medie
- Liceo o istituto professionale
- Laurea triennale
- Laurea magistrale
- Specializzazione/Master/Dottorato

1. In che anno ha avuto la diagnosi di Sclerosi Multipla?
2. Forma di malattia come definita dal suo neurologo

- Recidivante-Remittente (RR)
- Secondariamente-Progressiva (SP)
- Primariamente-Progressiva (PP)
- Non lo so

1. Nelle ultime due settimane, per quanti giorni ha provato poco interesse o piacere nel fare le cose?

- Mai o quasi mai
- Diversi giorni
- Più della metà dei giorni
- Quasi tutti i giorni

1. Nelle ultime due settimane, per quanti giorni si è sentito/a giù di morale, depresso/a o senza speranze?

- Mai o quasi mai
- Diversi giorni
- Più della metà dei giorni
- Quasi tutti i giorni

1. Attualmente ritiene di avere difficoltà di memoria?

- Si
- No

1. Attualmente ritiene di avere difficoltà di attenzione e/o concentrazione?

- Si
- No

Le TUE domande

*Ora le chiediamo di confrontare due risposte diverse alla stessa domanda, riguardante i temi più discussi tra medici e persone con la Sclerosi Multipla, e di scegliere quale delle due risposte preferisce.*

**"In questo periodo estivo mi sento più stanco, cosa posso prendere?"**

*Risposta 1:

Grazie per condividere le tue sensazioni con noi. È normale che le persone con sclerosi multipla possano sperimentare un aumento della stanchezza durante il periodo estivo a causa dell'aumento delle temperature. Per affrontare questa situazione, ti consiglio di considerare alcune precauzioni.

Assicurati di bere a sufficienza per evitare la disidratazione, che può peggiorare la fatica. Cerca di rimanere in ambienti freschi durante le giornate più calde e utilizza abiti leggeri. Organizza le tue attività in modo da evitare i momenti più caldi della giornata, se possibile. Continua a fare esercizio fisico, ma in momenti più freschi e con moderazione. Se la stanchezza diventa eccessiva o si accompagna a sintomi nuovi o preoccupanti, ti consiglio di fissare una visita con noi per escludere altre possibili cause.

Non esitare a contattarmi se hai ulteriori domande o se desideri discutere ulteriormente la tua situazione.

Risposta 2:

Purtroppo, alcuni pazienti affetti da sclerosi multipla possono percepire una maggiore stanchezza nei periodi più caldi così come in quelli più freddi dell’anno. In questi casi, è importante evitare di uscire nelle ore più calde, esporsi al sole senza bagnarsi ed è essenziale garantire un’adeguata idratazione.

Se nonostante questi accorgimenti il sintomo però dovesse persistere e/o essere tale da compromettere le sue attività fisiche o la sua vita sociale, lavorativa e familiare, allora sarebbe utile una visita neurologica per l’eventuale introduzione di una terapia sintomatica.

- Preferisco la Risposta 1
- Preferisco la Risposta 2

1. Indichi in una scala da 1 a 5 quanto le è piaciuta la Risposta n°1

- 1
- 2
- 3
- 4
- 5

1. Indichi in una scala da 1 a 5 quanto le è piaciuta la Risposta n°2

- 1
- 2
- 3
- 4
- 5

**“Dottore ho fatto la Risonanza Magnetica e c’è una nuova lesione, che devo fare?”**

Risposta 1:

Innanzitutto, cerchiamo di concordare una data per una rivalutazione clinica. In tale occasione possiamo rivedere le immagini del suo esame ed effettuare una visita clinica. Soltanto dopo l’integrazione di queste informazioni potremo discutere eventualmente della necessità o meno di un cambio della terapia, qualora valutassimo non pienamente soddisfacente l’efficacia della terapia in corso. La comparsa di una nuova lesione alla Risonanza Magnetica, infatti, deve essere inquadrata nell’ambito di una serie di dati, tra cui la sua storia clinica, la distanza dall’inizio della terapia in corso nonché sulle caratteristiche della lesione. Ad ogni modo, non si preoccupi perché, anche qualora dovessimo cambiare terapia, abbiamo molte opzioni terapeutiche a nostra disposizione.

*Risposta 2:

Grazie per averci informato dei risultati della sua ultima risonanza magnetica. Capisco che la scoperta di una nuova lesione possa destare preoccupazione e ansia. Voglio assicurarle che siamo qui per supportarla e valutare attentamente la situazione.

La comparsa di una nuova lesione nella Sclerosi Multipla può essere un segno di attività della malattia. È importante che valutiamo insieme la situazione clinicamente per determinare il miglior percorso da seguire.

La preghiamo di prenotare un appuntamento il prima possibile, in modo che possiamo esaminare attentamente i risultati della risonanza magnetica e discutere delle opzioni di trattamento disponibili. In base alla valutazione clinica e ai suoi sintomi, potremo considerare opportune modifiche al suo attuale piano terapeutico o valutare l'adozione di nuove strategie per gestire la sua condizione.

Ricordi che, nonostante la presenza di una nuova lesione, ogni paziente reagisce in modo diverso e ciò non deve essere causa di allarmismo. Siamo qui per affrontare insieme questa fase e adattare il trattamento in modo da garantirle il miglior controllo possibile della Sclerosi Multipla.

Non esiti a contattarci se ha qualsiasi altra domanda o preoccupazione. Siamo a disposizione per fornirle tutto il supporto e l'assistenza di cui ha bisogno.

- Preferisco la Risposta 1
- Preferisco la Risposta 2

1. Indichi in una scala da 1 a 5 quanto le è piaciuta la Risposta n°1

- 1
- 2
- 3
- 4
- 5

1. Indichi in una scala da 1 a 5 quanto le è piaciuta la Risposta n°2

- 1
- 2
- 3
- 4
- 5

**"Nell’ultimo periodo sento che mi stanco più facilmente quando cammino per lunghi tratti, è una ricaduta?"**

*Risposta 1:

Grazie per condividere le tue preoccupazioni. Comprendo quanto sia importante monitorare attentamente la tua salute nella gestione della sclerosi multipla. La stanchezza più marcata durante la camminata potrebbe avere diverse cause, non necessariamente una ricaduta. Le ricadute sono solitamente caratterizzate da nuovi sintomi o da un significativo peggioramento di quelli esistenti per almeno 24 ore.

Per comprendere meglio la situazione, ti consiglio di tenere un registro dei tuoi sintomi e annotare quando avverti questa fatica. Se noti altri sintomi nuovi o un peggioramento di quelli esistenti, ti prego di contattarmi direttamente o di fissare un appuntamento nel nostro centro per una valutazione più approfondita.

Nel frattempo, è importante che tu presti attenzione al tuo benessere generale, riposi adeguatamente e faccia attività fisica in base alle tue capacità. Se la stanchezza persiste o si aggrava, potremmo discutere di strategie per gestirla in modo più efficace.

Resto a tua disposizione per qualsiasi ulteriore informazione o per pianificare una visita di follow-up.

Risposta 2:

Che ne dice di anticipare la visita di controllo così che possiamo effettuare una rivalutazione clinica? In questo modo potremo effettuare un confronto con la sua visita precedente e ripetere anche alcuni test, già eseguiti in passato, che ci permetteranno di valutare se vi è stata effettivamente una progressione della sua condizione clinica o se magari ci sono altri aspetti che possono contribuire a tale sintomo. Tenga presente che la stanchezza è un sintomo comune nei pazienti affetti da sclerosi multipla e che può presentarsi con una intensità talvolta lieve talvolta severa senza necessariamente configurare una ricaduta clinica. Inoltre, molti altri fattori possono contribuire all’ acuirsi del sintomo (ad esempio le temperature estreme, una scarsa idratazione, altre patologie concomitanti). Proprio al tal proposito, le chiederei, in accordo col suo medico curante di fare intanto delle analisi del sangue, un emocromo, la sideremia, gli elettroliti sierici ed una valutazione cardiologica per escludere cause frequenti di astenia (ad esempio una cardiopatia o anche eventuale iper/ipotensione).

Qualora a seguito di tali indagini e della nostra valutazione clinica dovessimo evidenziare una progressione clinica della sclerosi multipla faremo un’opportuna rivalutazione terapeutica.

- Preferisco la Risposta 1
- Preferisco la Risposta 2

1. Indichi in una scala da 1 a 5 quanto le è piaciuta la Risposta n°1

- 1
- 2
- 3
- 4
- 5

1. Indichi in una scala da 1 a 5 quanto le è piaciuta la Risposta n°2

- 1
- 2
- 3
- 4
- 5

**"Dottore il mio medico di base mi ha prescritto l’antibiotico per un’infezione urinaria. Posso prenderlo?"**

Risposta 1:

Sì, in generale può assumere la terapia antibiotica prescritta dal medico di base che l’ha visitata per questo problema. Per una migliore gestione è importante comunque scegliere l’antibiotico più adeguato al ceppo batterico responsabile dell’infezione, praticando sempre prima un’urinocoltura con antibiogramma. Chiaramente è importante anche che lei informi il suo medico di base che ha prescritto l’antibiotico, della terapia che sta assumendo per la Sclerosi Multipla per evitare eventuali interazioni. Per questo, le chiedo di tenermi informato/a relativamente al decorso dell’infezione e di riferirmi se tali episodi di infezioni delle vie urinarie diventassero ricorrenti, perché, in tal caso, dovremmo indagare sulle possibili cause, eventualmente correlate alla Sclerosi Multipla o alla sua terapia.

*Risposta 2:

Grazie per la tua email. È importante affrontare prontamente un'infezione urinaria, ma dobbiamo fare attenzione data la tua condizione di sclerosi multipla. Puoi sicuramente prendere l'antibiotico prescritto dal tuo medico di base, ma per garantire la tua sicurezza, ti prego di fornirmi il nome del farmaco e qualsiasi altro farmaco che stai assumendo per la sclerosi multipla. In questo modo, posso verificare eventuali interazioni farmacologiche o controindicazioni specifiche alla tua situazione medica.

Nel frattempo, se l'infezione urinaria è dolorosa o persiste, ti consiglio di contattare il tuo medico di base per ottenere ulteriori dettagli e istruzioni sulla terapia antibiotica. Siamo qui per supportarti e assicurarci che ricevi il miglior trattamento possibile, tenendo sempre presente la tua salute complessiva.

Resto a tua disposizione per ulteriori domande o preoccupazioni.

- Preferisco la Risposta 1
- Preferisco la Risposta 2

1. Indichi in una scala da 1 a 5 quanto le è piaciuta la Risposta n°1

- 1
- 2
- 3
- 4
- 5

1. Indichi in una scala da 1 a 5 quanto le è piaciuta la Risposta n°2

- 1
- 2
- 3
- 4
- 5

Adesso le riproponiamo una delle domande precedenti con le sue due risposte.

1. Come giudica la Risposta 1 nel farla sentire a suo agio (nell’essere cordiale e accogliente verso di lei, nel trattarla con rispetto, senza essere freddo e brusco)?

- Scarsa
- Accettabile
- Buona
- Più che buona
- Eccellente
- Non applicabile

1. Come giudica la Risposta 1 nel lasciarle raccontare la sua “storia” (Nel darle il tempo per descrivere pienamente il suo problema con parole sue; senza interrompere, senza farle fretta e senza distrarla)

- Scarsa
- Accettabile
- Buona
- Più che buona
- Eccellente
- Non applicabile

1. Come giudica la Risposta 1 nell’ascoltarla per davvero (Nel prestare molta attenzione a quanto stava dicendo; senza distrarsi guardando gli appunti o il computer mentre lei stava parlando)

- - Scarsa
- Accettabile
- Buona
- Più che buona
- Eccellente
- Non applicabile

1. Come giudica la Risposta 1 nell'essere interessata a lei come persona (nel chiedere/venire a conoscenza di importanti dettagli della sua vita, della sua situazione; senza trattarla semplicemente come un “numero”)?

- Scarsa
- Accettabile
- Buona
- Più che buona
- Eccellente
- Non applicabile

1. Come giudica la Risposta 1 nel comprendere pienamente le sue preoccupazioni (nel comunicare di aver pienamente compreso le sue preoccupazioni, senza ignorare o minimizzare nulla)?

- Scarsa
- Accettabile
- Buona
- Più che buona
- Eccellente
- Non applicabile

1. Come giudica la Risposta 1 nel dimostrare premura e vicinanza (nel mostrarsi sinceramente interessato alla sua situazione, nel rapportarsi con lei con umanità; senza essere indifferente o “distaccato”)?

- Scarsa
- Accettabile
- Buona
- Più che buona
- Eccellente
- Non applicabile

1. Come giudica la Risposta 1 nell'avere un atteggiamento positivo (nell’avere un comportamento positivo; essendo chiaro, senza atteggiamenti negativi circa i suoi problemi)?

- Scarsa
- Accettabile
- Buona
- Più che buona
- Eccellente
- Non applicabile

1. Come giudica la Risposta 1 nello spiegare le cose chiaramente (nel rispondere pienamente alle sue domande; nel dare spiegazioni chiare; nel dare informazioni adeguate; senza essere vago)?

- Scarsa
- Accettabile
- Buona
- Più che buona
- Eccellente
- Non applicabile

1. Come giudica la Risposta 1 nell'aiutarla ad assumere il controllo (nell’esplorare con lei le sue capacità di migliorare autonomamente la sua salute; nell’incoraggiarla a trovare soluzioni invece di dirle cosa deve fare)?

- Scarsa
- Accettabile
- Buona
- Più che buona
- Eccellente
- Non applicabile

1. Come giudica la Risposta 1 nel creare un piano di azione con lei (nel discutere le opzioni, nel coinvolgerla nelle decisioni per quanto desidera, senza ignorare il suo punto di vista)?

- Scarsa
- Accettabile
- Buona
- Più che buona
- Eccellente
- Non applicabile

1. Come giudica la Risposta 2 nel farla sentire a suo agio (nell’essere cordiale e accogliente verso di lei, nel trattarla con rispetto, senza essere freddo e brusco)?

- Scarsa
- Accettabile
- Buona
- Più che buona
- Eccellente
- Non applicabile

1. Come giudica la Risposta 2 nel lasciarle raccontare la sua “storia” (Nel darle il tempo per descrivere pienamente il suo problema con parole sue; senza interrompere, senza farle fretta e senza distrarla)

- Scarsa
- Accettabile
- Buona
- Più che buona
- Eccellente
- Non applicabile

1. Come giudica la Risposta 2 nell’ascoltarla per davvero (Nel prestare molta attenzione a quanto stava dicendo; senza distrarsi guardando gli appunti o il computer mentre lei stava parlando)

- - Scarsa
- Accettabile
- Buona
- Più che buona
- Eccellente
- Non applicabile

1. Come giudica la Risposta 2 nell'essere interessata a lei come persona (nel chiedere/venire a conoscenza di importanti dettagli della sua vita, della sua situazione; senza trattarla semplicemente come un “numero”)?

- Scarsa
- Accettabile
- Buona
- Più che buona
- Eccellente
- Non applicabile

1. Come giudica la Risposta 2 nel comprendere pienamente le sue preoccupazioni (nel comunicare di aver pienamente compreso le sue preoccupazioni, senza ignorare o minimizzare nulla)?

- Scarsa
- Accettabile
- Buona
- Più che buona
- Eccellente
- Non applicabile

1. Come giudica la Risposta 2 nel dimostrare premura e vicinanza (nel mostrarsi sinceramente interessato alla sua situazione, nel rapportarsi con lei con umanità; senza essere indifferente o “distaccato”)?

- Scarsa
- Accettabile
- Buona
- Più che buona
- Eccellente
- Non applicabile

1. Come giudica la Risposta 2 nell'avere un atteggiamento positivo (nell’avere un comportamento positivo; essendo chiaro, senza atteggiamenti negativi circa i suoi problemi)?

- Scarsa
- Accettabile
- Buona
- Più che buona
- Eccellente
- Non applicabile

1. Come giudica la Risposta 2 nello spiegare le cose chiaramente (nel rispondere pienamente alle sue domande; nel dare spiegazioni chiare; nel dare informazioni adeguate; senza essere vago)?

- Scarsa
- Accettabile
- Buona
- Più che buona
- Eccellente
- Non applicabile

1. Come giudica la Risposta 2 nell'aiutarla ad assumere il controllo (nell’esplorare con lei le sue capacità di migliorare autonomamente la sua salute; nell’incoraggiarla a trovare soluzioni invece di dirle cosa deve fare)?

- Scarsa
- Accettabile
- Buona
- Più che buona
- Eccellente
- Non applicabile

1. Come giudica la Risposta 2 nel creare un piano di azione con lei (nel discutere le opzioni, nel coinvolgerla nelle decisioni per quanto desidera, senza ignorare il suo punto di vista)?

- Scarsa
- Accettabile
- Buona
- Più che buona
- Eccellente
- Non applicabile

**Full version of the form (translated English version)**

*For the sake of clarity, in this translated version, we have indicated with an asterisk the responses provided by ChatGPT for the reviewer’s attention.*

*The four questions were presented to both Neurologists and ChatGPT with the following prompt: "How would you respond to a multiple sclerosis patient asking..."*

Demographics

1. Sex

- Male
- Female
- Other
- Prefer not to say

1. Year of birth
2. Area of residence

- Abruzzo
- Basilicata
- Calabria
- Campania
- Emilia-Romagna
- Friuli-Venezia Giulia
- Lazio
- Liguria
- Lombardia
- Marche
- Molise
- Piemonte
- Puglia
- Sardegna
- Sicilia
- Toscana
- Trentino-Alto Adige
- Umbria
- Valle d’Aosta
- Veneto

1. Educational level

- Elementary school
- Middle school
- High school or technical school
- Bachelor's degree
- Master's degree
- Specialization/Ph.D.

1. In what year were you diagnosed with Multiple Sclerosis?
2. Type of MS as defined by your neurologist

- Relapsing-Remitting (RR)
- Secondary Progressive (SP)
- Primary Progressive (PP)
- I don't know

1. Over the last 2 weeks, how often have you been bothered by: having little interest or pleasure in doing things?

- Not at all
- Several days
- More than half the days
- Nearly every day

1. Over the last 2 weeks, how often have you been bothered by: feeling down, depressed or hopeless?

- Not at all
- Several days
- More than half the days
- Nearly every day

1. Are you currently experiencing any memory difficulties?

- Yes
- No

1. Are you currently experiencing any difficulties with attention and/or concentration?

- Yes
- No

YOUR questions

*Now we ask you to compare two different responses to the same question, regarding the most discussed topics among doctors and people with Multiple Sclerosis and choose which of the two responses you prefer.*

**"** **I feel more tired during summer season, what shall I do?"**

*Response 1:

Thank you for sharing your concerns with us. It's normal for people with multiple sclerosis to experience increased fatigue during the summer due to rising temperatures. To address this situation, I recommend considering some precautions.

Make sure to drink enough to avoid dehydration, which can exacerbate fatigue. Try to stay in cooler environments during the hottest parts of the day and wear lightweight clothing. Plan your activities to avoid the hottest times of the day, if possible. Continue to exercise, but do so in cooler moments and in moderation. If the fatigue becomes excessive or is accompanied by new or concerning symptoms, I recommend scheduling a visit with us to rule out other possible causes.

Please don't hesitate to contact me if you have any further questions or if you would like to discuss your situation further.

Response 2:

Unfortunately, some people with multiple sclerosis may experience increased fatigue during both hot and cold periods of the year. In such cases, it's important to avoid going out during the heat hours, avoid prolonged sun exposure, and ensure proper hydration.

If, despite these precautions, the symptom persists and interferes with your physical activities or your social, work, and family life, then a neurological evaluation for the possible introduction of symptomatic therapy would be useful.

- I prefer Response 1.
- I prefer Response 2.

1. Rate how much you liked Response 1 on a scale from 1 to 5:

- 1
- 2
- 3
- 4
- 5

1. Rate how much you liked Response 2 on a scale from 1 to 5:

- 1
- 2
- 3
- 4
- 5

**"** **I have had new brain MRI, and there is one new lesion. What should I do?"**

Response 1:

First, let's schedule a new clinical consultation. During the appointment, we can review the images from your MRI and have a clinical examination. Only after integrating this information we can discuss the potential need to change your therapy if we find the current treatment's effectiveness to be unsatisfactory. The appearance of a new lesion on the MRI should be considered within the context of various data, including your clinical history, the duration of your current therapy, and the characteristics of the lesion. However, please don't worry because even if we need to change the therapy, we have many therapeutic options available.

*Response 2:

Thank you for informing us about the results of your recent MRI. I understand that discovering a new lesion can be concerning and anxiety-inducing. I want to assure you that we are here to support you and carefully evaluate the situation.

The appearance of a new lesion in Multiple Sclerosis can be a sign of disease activity. It is important for us to assess the situation clinically to determine the best course of action.

Please book an appointment as soon as possible so that we can thoroughly examine the MRI results and discuss the available treatment options. Based on the clinical evaluation and your symptoms, we may consider appropriate modifications to your current treatment plan or explore new strategies to manage your condition.

Remember that, despite the presence of a new lesion, every patient reacts differently, and this should not cause alarm. We are here to face this phase together and adjust the treatment to ensure the best possible control of Multiple Sclerosis.

Do not hesitate to contact us if you have any other questions or concerns. We are here to provide you with all the support and assistance you need.

- I prefer Response 1.
- I prefer Response 2.

1. Rate how much you liked Response 1 on a scale from 1 to 5:

- 1
- 2
- 3
- 4
- 5

1. Rate how much you liked Response 2 on a scale from 1 to 5:

- 1
- 2
- 3
- 4

**“Recently, I’ve been feeling tired more easily when walking long distances. Am I relapsing?”**

*Response 1:

Thank you for sharing your concerns. I understand how important it is to carefully monitor your health in the management of multiple sclerosis. Increased fatigue during walking could have various causes, and do not necessarily represent a relapse. Relapses are typically characterized by new symptoms or a significant worsening of existing ones for at least 24 hours. To better understand the situation, I recommend keeping a record of your symptoms and noting when you experience this fatigue. If you notice any new symptoms or a worsening of existing ones, please contact me directly or schedule an appointment at our center for a more in-depth evaluation. In the meantime, it's important for you to pay attention to your overall well-being, get adequate rest, and engage in physical activity according to your abilities. If the fatigue persists or worsens, we can discuss strategies for managing it more effectively. I am here for any further information or to plan a follow-up visit.

Response 2:

What do you think about moving up your follow-up appointment so that we can perform a clinical evaluation? This way, we can compare it with your previous examination and repeat some previously conducted tests, allowing us to evaluate whether there has been an actual progression of your clinical condition or if there may be other aspects contributing to this symptom. Keep in mind that fatigue is a common symptom in patients with multiple sclerosis and can vary in intensity, sometimes mild, sometimes severe, without necessarily constituting a clinical relapse. Additionally, many other factors can contribute to the worsening of the symptom (such as extreme temperatures, dehydration, and other concurrent medical conditions). In this regard, I would ask you, in consultation with your primary care physician, to undergo blood tests, a complete blood count, serum iron levels, serum electrolytes, and a cardiological evaluation to rule out common causes of fatigue (such as heart disease or potential hypertension). If, following these tests and our clinical evaluation, we detect a clinical progression of multiple sclerosis, we will conduct an appropriate therapeutic reevaluation.

- I prefer Response 1.
- I prefer Response 2.

1. Rate how much you liked Response 1 on a scale from 1 to 5:

- 1
- 2
- 3
- 4
- 5

1. Rate how much you liked Response 2 on a scale from 1 to 5:

- 1
- 2
- 3
- 4

**“My primary care physician has given me a prescription for an antibiotic for a urinary infection. Is there any contraindication for me?”**

Response 1:

Yes, in general, you can take the antibiotic therapy prescribed by your primary care physician for this issue. For better management, it's important to choose the antibiotic that is most appropriate for the bacterial strain responsible for the infection, always starting with a urine culture and sensitivity test. Of course, it's also important that you inform your primary care physician, who prescribed the antibiotic, about the therapy you are taking for Multiple Sclerosis to avoid any potential interactions. For this reason, please keep me informed about the progress of the infection and let me know if these urinary tract infection episodes become recurrent, as, in such cases, we should investigate the possible causes, potentially related to Multiple Sclerosis or its treatment.

*Response 2:

Thank you for your email. It's important to address a urinary tract infection promptly, but we need to be cautious given your condition of multiple sclerosis. You can certainly take the antibiotic prescribed by your primary care physician, but to ensure your safety, please provide me with the name of the medication and any other medication you are taking for multiple sclerosis. This way, I can check for potential drug interactions or specific contraindications related to your medical condition. In the meantime, if the urinary tract infection is painful or persists, I recommend contacting your primary care physician to get further details and instructions on antibiotic therapy. We are here to support you and ensure you receive the best possible treatment, always considering your overall health.

- I prefer Response 1.
- I prefer Response 2.

1. Rate how much you liked Response 1 on a scale from 1 to 5:

- 1
- 2
- 3
- 4
- 5

1. Rate how much you liked Response 2 on a scale from 1 to 5:

- 1
- 2
- 3
- 4
- 5

Now we'll present one of the previous questions with its two responses

*(Author note for the reviewer: this section was repeated for each of the four questions* *along with their respective two responses)*

1. How do you rate Response 1 in making you feel at ease (introducing him/herself, explaining his/her position, being friendly and warm towards you, treating you with respect; not cold or abrupt)

- Poor
- Fair
- Good
- Very good
- Excellent
- Does not apply

1. How do you rate Response 1 in letting you tell your "story" (giving you time to fully describe your condition in your own words; not interrupting, rushing or diverting you)

- Poor
- Fair
- Good
- Very good
- Excellent
- Does not apply

1. How do you rate Response 1 in really listening (paying close attention to what you were saying; not looking at the notes or computer as you were talking)

- Poor
- Fair
- Good
- Very good
- Excellent
- Does not apply

1. How do you rate Response 1 in being interested in you as a whole person (asking/knowing relevant details about your life, your situation; not treating you as "just a number")

- Poor
- Fair
- Good
- Very good
- Excellent
- Does not apply

1. How do you rate Response 1 in fully understanding your concerns (communicating that he/she had accurately understood your concerns and anxieties; not overlooking or dismissing anything)

- Poor
- Fair
- Good
- Very good
- Excellent
- Does not apply

1. How do you rate Response 1 in showing care and compassion (seeming genuinely concerned, connecting with you on a human level; not being indifferent or "detached")

- Poor
- Fair
- Good
- Very good
- Excellent
- Does not apply

1. How do you rate Response 1 in being positive (having a positive approach and a positive attitude; being honest but not negative about your problems)

- Poor
- Fair
- Good
- Very good
- Excellent
- Does not apply

1. How do you rate Response 1 in explaining things clearly (fully answering your questions; explaining clearly, giving you adequate information; not being vague)

- Poor
- Fair
- Good
- Very good
- Excellent
- Does not apply

1. How do you rate Response 1 in helping you to take control (exploring with you what you can do to improve you health yourself; encouraging rather than "lecturing" you)

- Poor
- Fair
- Good
- Very good
- Excellent
- Does not apply

1. How do you rate Response 1 in making a plan of action with you (discussing the options, involving you in decisions as much as you want to be involved; not ignoring your views)

- Poor
- Fair
- Good
- Very good
- Excellent
- Does not apply

1. How do you rate Response 2 in making you feel at ease (introducing him/herself, explaining his/her position, being friendly and warm towards you, treating you with respect; not cold or abrupt)

- Poor
- Fair
- Good
- Very good
- Excellent
- Does not apply

1. How do you rate Response 2 in letting you tell your "story" (giving you time to fully describe your condition in your own words; not interrupting, rushing or diverting you)

- Poor
- Fair
- Good
- Very good
- Excellent
- Does not apply

1. How do you rate Response 2 in really listening (paying close attention to what you were saying; not looking at the notes or computer as you were talking)

- Poor
- Fair
- Good
- Very good
- Excellent
- Does not apply

1. How do you rate Response 2 in being interested in you as a whole person (asking/knowing relevant details about your life, your situation; not treating you as "just a number")

- Poor
- Fair
- Good
- Very good
- Excellent
- Does not apply

1. How do you rate Response 2 in fully understanding your concerns (communicating that he/she had accurately understood your concerns and anxieties; not overlooking or dismissing anything)

- Poor
- Fair
- Good
- Very good
- Excellent
- Does not apply

1. How do you rate Response 2 in showing care and compassion (seeming genuinely concerned, connecting with you on a human level; not being indifferent or "detached")

- Poor
- Fair
- Good
- Very good
- Excellent
- Does not apply

1. How do you rate Response 2 in being positive (having a positive approach and a positive attitude; being honest but not negative about your problems)

- Poor
- Fair
- Good
- Very good
- Excellent
- Does not apply

1. How do you rate Response 2 in explaining things clearly (fully answering your questions; explaining clearly, giving you adequate information; not being vague)

- Poor
- Fair
- Good
- Very good
- Excellent
- Does not apply

1. How do you rate Response 2 in helping you to take control (exploring with you what you can do to improve you health yourself; encouraging rather than "lecturing" you)

- Poor
- Fair
- Good
- Very good
- Excellent
- Does not apply

1. How do you rate Response 2 in making a plan of action with you (discussing the options, involving you in decisions as much as you want to be involved; not ignoring your views)

- Poor
- Fair
- Good
- Very good
- Excellent
- Does not apply

**Patient Health Questionnaire-2 (PHQ2)**

The occurrence of depressive symptoms and mood deflection was surveyed using the Patient Health Questionnaire (PHQ)-2 scale. PHQ-2 includes the first two items of the PHQ-9 (over the last 2 weeks, how often have you been bothered by the following problems? 1. Little interest or pleasure in doing things; 2. Feeling down, depressed or hopeless). Final scores range from 0 to 6, whereby values above 3 are strongly indicative of a possible depressive condition. The purpose of PHQ-2 is not to provide definite diagnosis, but to screen for depression using a "first-step" approach.

**Consultation and Relational Empathy (CARE)**

The Consultation and Relational Empathy (CARE) scale was employed to assess the perceived empathy of PwMS on the different responses. This scale measures empathy within the context of the doctor-patient relationship, and and consists of ten questions, each rated by 5 levels (1 - poor; 2 - fair; 3 - good; 4 - very good; 5 - excellent). Upon completion, the ten answers are added together to obtain a maximum score of 50 and a minimum score of 10. Up to two “Does not apply” responses or missing values are allowable, and are replaced with the average score for the remaining items.

|  | **Total**  **(n=34)** | **Group A (n=5)** | **Group B**  **(n=19)** | **Group C (n=5)** | **Group D (n=5)** |
| --- | --- | --- | --- | --- | --- |
| **Age in years, mean (SD)** | 42.47 (10.65) | 40.8 (11.10) | 43.32 (10.03) | 41.2 (10.35) | 42.2 (15.58) |
| **Female, n (%)** | 20 (58.82%) | 3 (60) | 11 (58.9) | 3 (60) | 3 (60) |
| **Clinical practice in neurology in years, mean (SD)** | 12.94 (9.78) | 12 (10.25) | 13.32 (9.18) | 12 (10.1) | 13.4 (14.15) |

**Table 1. Demographic and clinical characteristics of the participant neurologists (N=34).** Note. (n): number; SD: standard deviation.


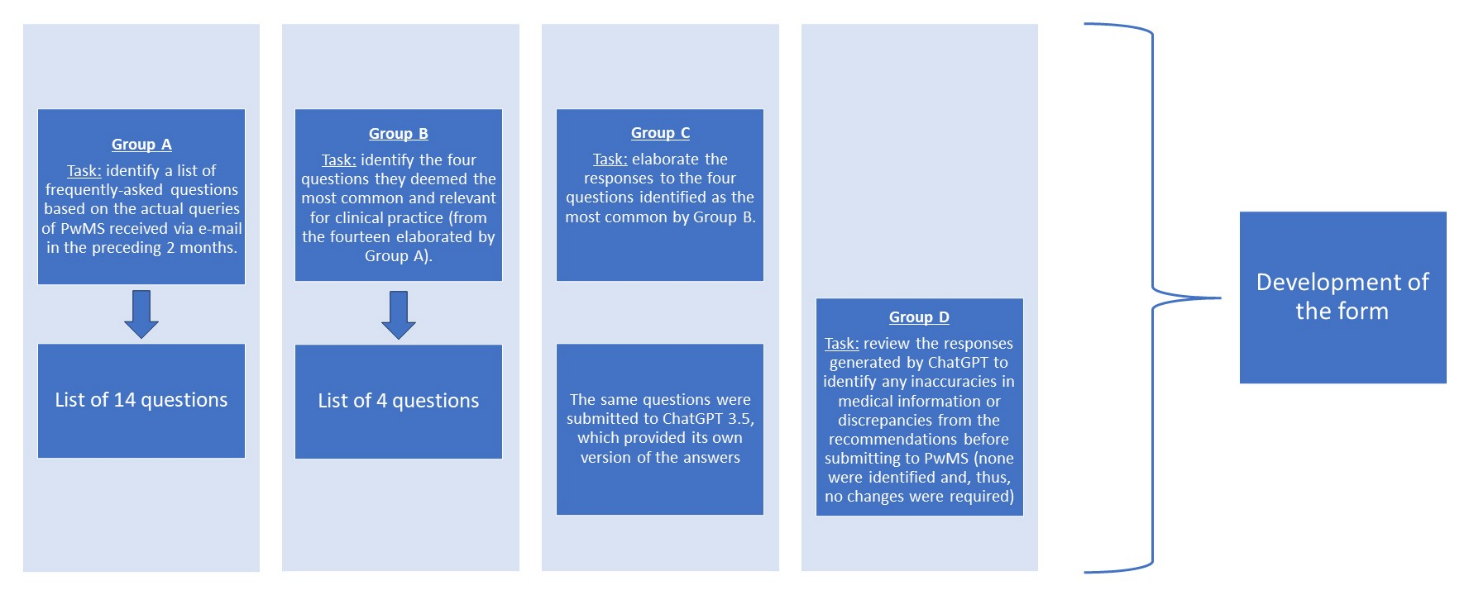


**Figure 1. Workflow process.** The image shows the workflow that led to the completion of the form. The 34 participating neurologists were randomly assigned to four working groups using Research Randomizer, an online software.
